# Detection of Human Circulating and Extracellular Vesicle-Derived miRNAs in Serum of Humanized Mice Transplanted with Human Breast Cancer (HER2^+^ and TNBC) Cells—A Proof of Principle Investigation

**DOI:** 10.3390/ijms26083629

**Published:** 2025-04-11

**Authors:** I-Peng Chen, Stefan Henning, Marc Bender, Sarah Degenhardt, Mouna Mhamdi Ghodbani, Ann Kathrin Bergmann, Beate Volkmer, Gero Brockhoff, Anja K. Wege, Rüdiger Greinert

**Affiliations:** 1 Department of Molecular Cell Biology, Skin Cancer Center Buxtehude, Elbekliniken Stade-Buxtehude, 21614 Buxtehude, Germany; i-peng.chen@elbekliniken.de (I.-P.C.); stefan.henning@elbekliniken.de (S.H.); marc.bender@elbekliniken.de (M.B.); mouna.mhamdi-ghodbani@elbekliniken.de (M.M.G.); beate.volkmer@elbekliniken.de (B.V.); 2 Core Facility of Electron Microscopy, University Clinics Duesseldorf, 40225 Duesseldorf, Germany; kathrin.bergmann@uni-duesseldorf.de; 3 Department of Gynecology and Obstetrics, Medical Center Regensburg, 93053 Regensburg, Germany; gero.brockhoff@ukr.de (G.B.); anja.wege@ukr.de (A.K.W.); 4 Bavarian Cancer Research Center (BZKF), 93053 Regensburg, Germany

**Keywords:** humanized tumor mouse, liquid biopsy, miRNA, extracellular vesicles, breast cancer

## Abstract

Humanized tumor mice (HTM) allow for preclinical cancer treatment studies of breast cancer (BC) under human-like conditions. This study utilized HTM for the first time to investigate potential miRNA biomarker candidates for treatment response in sera and extracellular vesicles (EVs), following X-irradiation and atezolizumab (anti-PD-L1) treatment. We identified the changes of human-specific miRNAs (miR-23b-3p and miR-155-5p) after irradiation and anti-PD-L1 treatment in HTMs with human epidermal growth factor receptor 2 positive (HER2^+^ BC) and triple-negative breast cancer (TNBC). The high degree of conserved, circulating free miRNA in mice and men represents a challenge of our assay; however, miRNAs with ≥2 nucleotide mismatches can be employed for human-specific analysis, and even conserved miRNAs may be utilized under clearly defined conditions of human tumor growth in HTM. A comparative analysis of extracellular vesicle miRNA cargo and free-circulating serum miRNAs revealed several exosome-specific miRNAs (miR-29b-3p, miR-34c-5p, miR-203a-3p, miR-378g, and miR-382-5p) in HTMs, which are known to play roles in BC. Our findings demonstrate that HTMs are a suitable model to identify treatment-induced changes in free-circulating and exosomal miRNAs that influence tumor progression and immunological tumor defense, both locally and at distant sites. This study presents a proof-of-principle approach to analyzing cell-free nucleotides and exosomes in a human-like, preclinical in vivo setting. Further refinements are necessary to enhance the sensitivity and the specificity of the HTM-based approach.

## 1. Introduction

Breast cancer (BC) remains the most prevalent malignant disease in women worldwide, with over 2.3 million new cases reported globally in 2022 [1]. Among the heterogeneous BC subtypes, triple-negative BC (TNBC) and HER2^+^ BC are recognized as the most aggressive forms, associated with poor prognosis [2]. TNBC, characterized by the absence of estrogen receptors (ER), progesterone receptors (PR), and HER2 expression [3], accounts for about 15–20% of all BC cases [4]. Conversely, HER2^+^ BC, which constitutes about 15% of BC cases [2,5], is defined by the overexpression of HER2, typically resulting from ERBB2 gene amplification.

While targeted therapies exist for HER2^+^ BC [2,6,7], treatment modalities for the highly immunogenic TNBC remain limited, despite advancements in immunotherapy [8,9,10,11,12,13]. Tumor cell elimination, tumor-immune cell equilibrium, and the tumor cell escape are determined by tumor-immune cell interactions [14]. Highly relevant for tumor cell escape is the PD-L1/PD-1 immune checkpoint axis [15,16,17], which curbs or even fully inhibits T-cell activity against tumor cells [18]. Aberrant PD-L1 has been observed in 50% of BCs [19], while TNBC cells show the most pronounced PD-L1 levels [20]. High levels of tumor-infiltrating lymphocytes (TILs), a high tumor mutational burden, and increased expression of PD-L1 render TNBC patients susceptible to immune checkpoint inhibitor (ICI) therapies, mainly PD-1- or PD-L1-directed immune modulations [20,21,22,23]. ICIs targeting the PD-1/PD-L1 axis have achieved notable response rates in TNBC patients, especially when applied in combination with chemotherapy, making immunotherapy a new therapeutic mainstay for the treatment of TNBC [12,17]. However, a substantial proportion of BC patients do not benefit from, or they develop resistance to, ICI therapies. [4,9,10,11,12].

This challenge extends to promising combinatorial approaches such as neoadjuvant radiotherapy and immunotherapy [24]. This novel therapy combination aims to elicit an immunological tumor defense by inducing localized tumor cell death through irradiation, while promoting systemic immune activation (abscopal effect). Unlike adjuvant irradiation, sublethal neoadjuvant application is expected to trigger the release of tumor cell-derived neoantigens, and to cause immunogenic (non-apoptotic) cell alterations and death. Preliminary studies highlight the potential of this approach [23,24], as reviewed by Debbi et al. [25]. However, therapeutically valid algorithms for a successful combination of radiation therapy and ICI (in terms of time point and sequence of treatment(s), applied doses, and fractionation regimens) are still missing [24]. Despite these advancements, robust prognostic or predictive biomarkers for successful therapy combinations involving radiotherapy and ICI remain elusive. Small non-coding RNAs, particularly circulating microRNAs (miRNAs), have emerged as promising candidates due to their alterations during tumor progression and their potential to predict and influence therapeutic outcomes.

miRNAs are small (~22 nt) non-coding oligonucleotides that epigenetically regulate gene expression by binding predominantly to the 3′UTR (and in some cases to 5′UTR and open reading frames) of target messenger RNA (mRNAs), inhibiting mRNA translation [26,27]. miRNA synthesis and function have been intensively reviewed elsewhere (e.g., in [28]). Its abnormal regulation is involved in carcinogenesis, as well as tumor growth and progression. Moreover, miRNAs have been found to be relevant actors in the regulation of immunological responses (including immune evasion) and the tumor microenvironment (TME) in BC patients [29,30]. Importantly, miRNAs can circulate in body fluids like plasma, serum, urine, etc., either as free molecules or as cargos of extracellular vesicles (e.g., exosomes) [31]. Specific changes of circulating miRNAs in body fluids (liquid biopsies) are correlated with BC diagnosis, tumor progression, and metastasis (e.g., [32,33,34,35]).

The role of circulating miRNAs in TNBC has been addressed [36,37,38], and several miRNAs with specific activities came into focus. For example, there is a 4-miRNA-panel (miR-19a, -20a, -126, and -155) which discriminates between early and metastasized BC. Moreover, low-expressed miR-155 in TNBC predicted a shorter disease-free survival [39], further indicating that circulating miRNAs are useful to monitor TNBC progression and therapy response [30,40]. Similar observations were recently summarized by Fogazzi et al. and Isca et al., who reviewed the role of miRNAs in HER2^+^ BC. This is especially the case for miRNA-21, the miRNA-125 family, miR-155, and miR-205, which seem to play a relevant role in BC development, diagnosis, prognosis, and prediction [5,41], although other miRNAs were also reported to have a considerable influence on the progression of HER2^+^ BC [42,43,44,45].

Attributing circulating miRNAs to their individual origins (tumor tissue, immune cells, tumor microenvironment, etc.) remains challenging. However, the detection, quantification, and identification of the miRNAs as the cargo of cell- or tumor-secreted extracellular vesicles (EVs), especially exosomes (small EVs, sEVs), offers a useful approach [34,46], as the frequency of circulating EVs and their cargo characteristics correlate with malignant disease occurrence and staging [47,48].

Exosomes, small EVs (50–150 nm diameter) surrounded by a lipid bilayer membrane, are generated and released by various cell types, including tumor cells. Importantly, during their biogenesis, exosomes are actively loaded with a molecular cargo of amino acids, DNA, proteins, metabolites, noncoding RNAs, like miRNAs, and other components [49,50]. Thus, the exosomal cargo serves as a fingerprint indicating the cell of origin. This is further supported by the membrane protein composition of the exosomal lipid bilayer, which is characterized by certain exosome-specific proteins, like the tetraspanins CD9, CD63, and CD81, but also by selected proteins, in which case the composition might be origin-specific. Based on their membrane protein composition, exosomes released by donor cells can actively be taken up (endocytosis/phagocytosis) by recipient cells in the micro- (and macro-) environment. Due to these capacities, exosomes are relevant players in intercellular communication, even across long distances [51,52]. Exosomes protect their cargo from degradation by encapsulating it in a lipid bilayer-shielded vesicular structure. Due to their stability, sEVs and their cargo can be monitored, and are therefore useful markers to follow processes associated with carcinogenesis, metastasis, and therapeutic response [53,54,55,56,57].

In many investigations, miRNA profiling was performed in tumor tissues instead of liquid biopsies (e.g., [58]). This procedure is inherently coupled with inconvenient invasive tissue biopsy sampling. Even more important, a tissue-based approach is unsuitable for longitudinal exploration of miRNA patterns as a function of treatment. Instead, the collection of free-circulating miRNAs or circulating sEVs-miRNAs by non-invasive liquid biopsies is much more appropriate to identify, monitor, and validate disease- and treatment-specific miRNAs. However, the translation of gained knowledge towards clinical exploitation is still pending and needs more preclinical in vivo-based approaches, for example, using human-like mouse models.

For this proof-of-principle approach, we utilized a Humanized Tumor Mouse (HTM) model to identify free-circulating miRNAs and exosomal miRNAs (sEV-miRNAs) in mouse blood sera. Due to the coexistence of human BC growth and a functional human immune system, HTM represents a human-like and treatment-relevant model. An miRNA analysis was performed in liquid biopsies (serum) derived from HTM transplanted with different BC types and treated with neoadjuvant irradiation and/or ICI. The high degree of conservation between miRNA sequences of mice and human origin (e.g., http://miRbase.org, [59,60]) presents a challenge for identifying human-specific miRNAs in xenograft models and HTM. Nevertheless, were able to discriminate between conserved miRNAs (of mice as well as human origin) and less-conserved miRNAs (1–2 mismatches in nucleotide sequence between mice and human), which represent “human specific” miRNA with 80–95% probability in our assay.

This study represents the first proof-of-principle report on detecting and analyzing microRNAs and sEVs-miRNAs (exosomal miRNA) on a humanized mouse background. We demonstrate that the HTM model is highly suitable for preclinical in vivo analyses of circulating (free) microRNAs and sEVs-miRNAs as a function of treatment and under human-like conditions.

## 2. Results

### Quantification of miRNA in Serum of Treated and Control Mice

In our first attempt to investigate whether we can detect circulating miRNAs in sera of human BC (HER2^+^, TNBC), cell line-transplanted mice (JIMT-1- and MDA-MB-231-transplanted HTM, see Table 1) under different treatment regimens (with and without irradiation and/or without anti-PD-L1 treatment), we analyzed circulating miRNAs in mice sera, as well as miRNAs from the exosomal fraction of sera, and compared the results to untreated controls and an NSG control (see Table 1).

**Figure 1:**
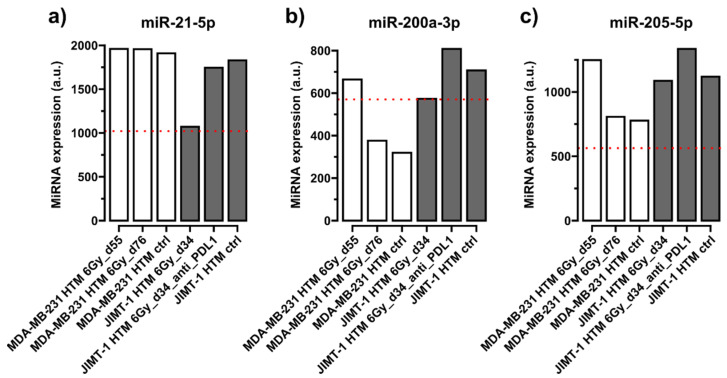
Expression levels of circulating **conserved miRNAs** in serum of HTM. Results from mice transplanted with MDA-MB-231 (TNBC) or with JIMT-1 (Her2^+^) are shown after different treatments (irradiation, 6 Gy, and 6 Gy + PD-L1 antibody, atezolizumab, [JIMT-1]) and compared to untreated HTM mice (ctrl), as well as to those miRNA expression levels in the serum of untreated non-humanized (without human immune cells) NSG mice (dotted red line). Time points after cell line transplantation are given in dxy. (**a**) **miR-21-5p**, (**b**) **miR-200a-3p**, (**c**) **miRNA-205-5p**. miRNA expression levels have been normalized to the geometrical mean of expression of the most stable miRNAs 16-5p, -22-3p, and -130a-3p in FirePlex Assay (see Section “The FirePlex^®^ Assay for miRNA Profiling in Serum and Exosome”). Expression values of miRNAs are given in arbitrary units (a.u.) of fluorescence intensity values in the flow cytometric FirePlex Assay.

**Figure 2:**
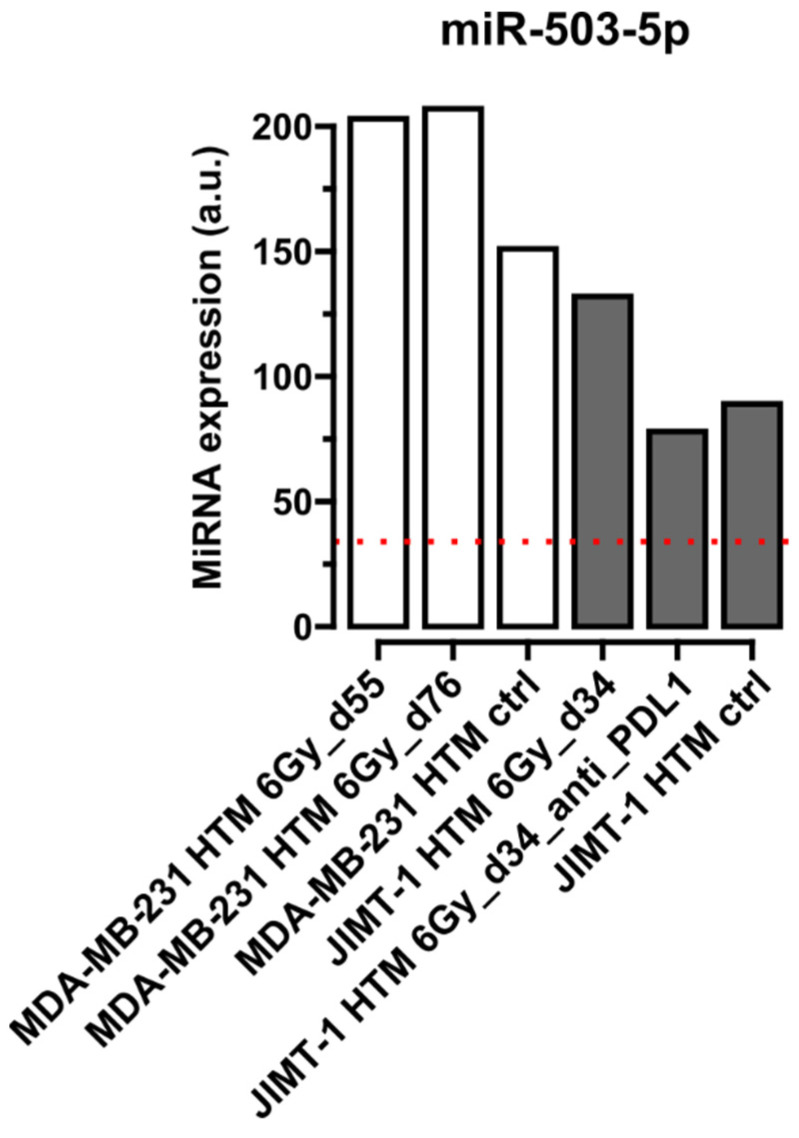
Expression level of circulating miR-503-5p (**one nucleotide mismatch** between mice and human miRNA sequence) in serum of HTM. Results from mice transplanted with TNBC cell line MDA-MB-231 and those transplanted with HER2^+^ cell line (JIMT-1) are shown after different treatments (irradiation, 6 Gy, and 6 Gy + PD-L1 antibody, azetolizumab, [JIMT-1]) and compared to untreated mice (ctrl), as well as to those miRNA expression levels in the serum of untreated non-humanized mice (NSG mice, dotted red line). Time points after cell line transplantation are given in dxy. miRNA expression level has been normalized to the geometrical mean of expression of the most stable miRNAs 16-5p, -22-3p, and -130a-3p in FirePlex Assay (see Section “The FirePlex^®^ Assay for miRNA Profiling in Serum and Exosome”). Expression values of miRNA are given in arbitrary units (a.u.) of fluorescence intensity values generated by the flow cytometric FirePlex Assay.

**Figure 3:**
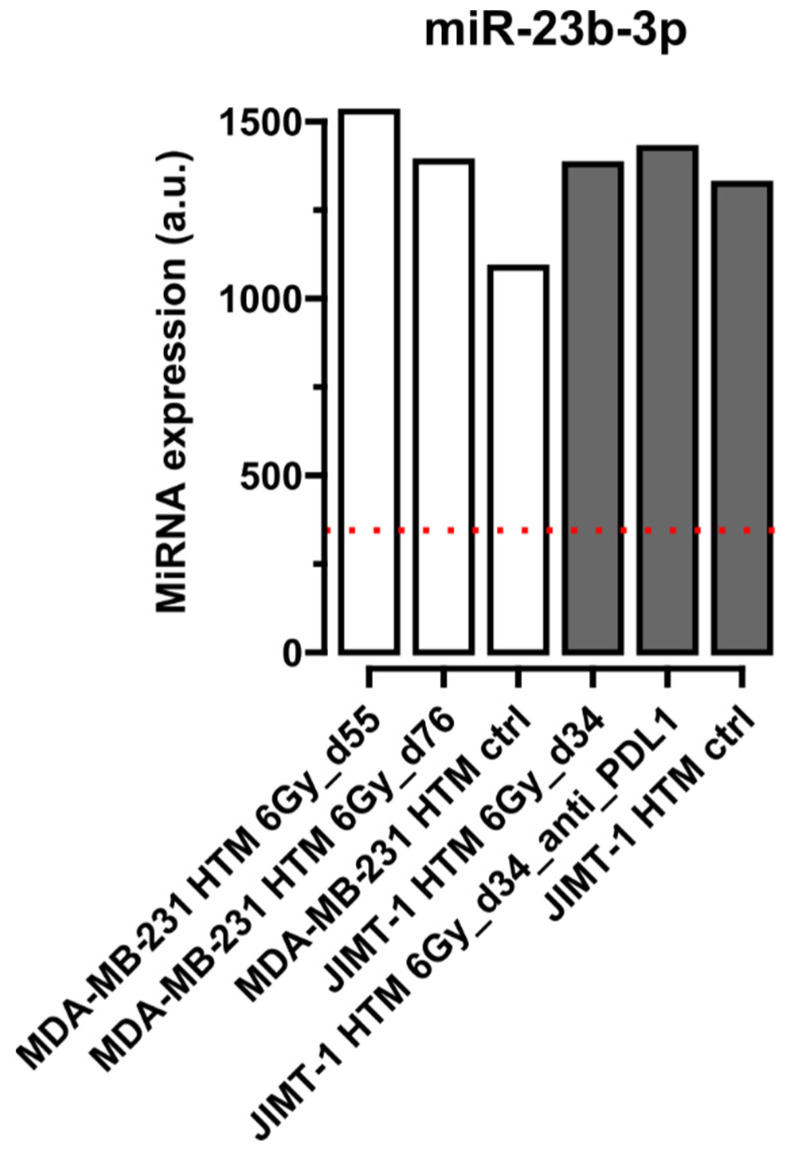
Expression level of circulating **miR-23b-3p** (**two nucleotide mismatches** between mice and human miRNA sequence) in serum of HTM. Results from mice transplanted with MDA-MB-231 (TNBC) or with JIMT-1 (HER2^+^) cell lines are shown after different treatments (irradiation, 6 Gy, and 6 Gy + anti-PD-L1). miRNA expression level for the selected miRNA is compared to the level in the serum of untreated non-humanized mice (NSG mice, dotted red line). Time points after cell line transplantation are given in dxy. miRNA has been normalized to the geometrical mean of expression of the most stable miRNAs, 16-5p, -22-3p, and -130a-3p, in FirePlex Assay (see Section “The FirePlex^®^ Assay for miRNA Profiling in Serum and Exosome”). Expression values of miRNA are given in arbitrary units (a.u.) of fluorescence intensity values in the flow cytometric FirePlex Assay.

**Figure 4:**
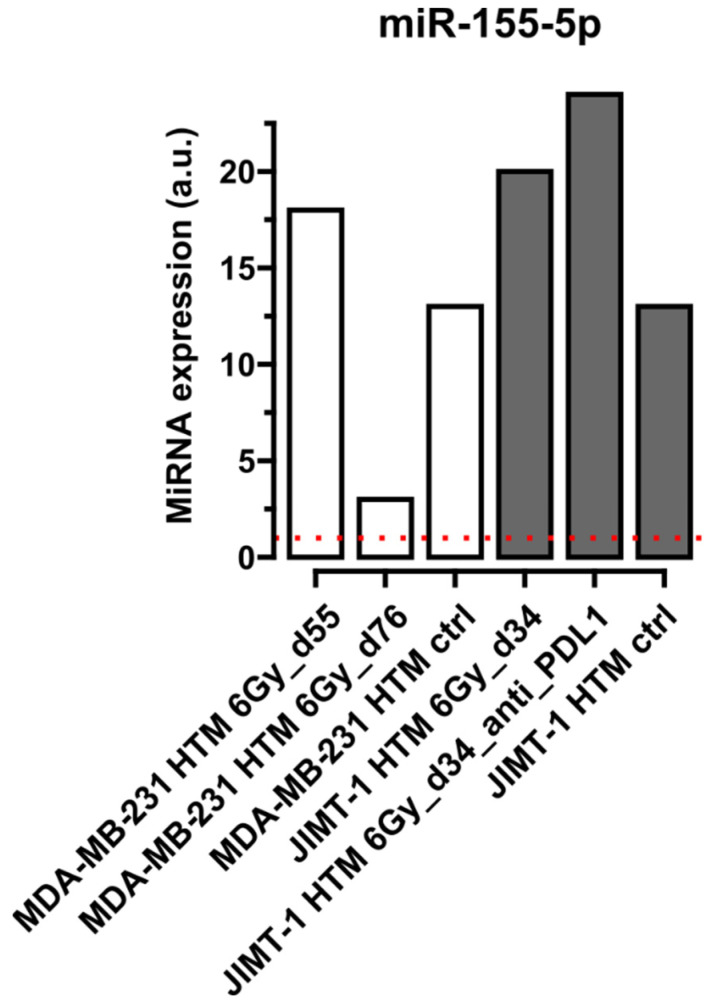
Circulating miR-155-5p (with two nucleotide mismatches between mice and human miRNA sequence, which is supposed to be of a specific human origin, according to [74]) in serum of HTM. Results from mice transplanted with MDA-MB-231(TNBC) or with JIMT1 (HER2^+^) cell lines are shown after different treatments (irradiation, 6 Gy, and 6 Gy + anti-PD-L1), compared to untreated mice (HTM ctrl). miRNA expression level for the selected miRNA is compared to its level in the serum of untreated non-humanized mice (NSG mice), dotted red line. Time points after cell line transplantation are given in dxy. miRNA expression has been normalized to geometrical mean of expression of the most stable miRNAs, 16-5p, -22-3p, and -130a-3p, in FirePlex Assay (see Section “The FirePlex^®^ Assay for miRNA Profiling in Serum and Exosome”). Expression values of miRNA are given in arbitrary units (a.u.) of fluorescence intensity values in the flow cytometric FirePlex Assay.

Figure 1, Figure 2, Figure 3 and Figure 4 show selected results for certain exemplary miRNAs in sera of HER2^+^ (JIMT-1 HTM) and TNBC (MDA-MB-231 HTM). Examples for the conserved miRNAs (Figure 1), one (Figure 2) and two nucleotide mismatche(s) between human and mice miRNA (Figure 3), as well as for a “human-specific” miRNA (Figure 4), are presented. Results for the expression of other miRNAs (listed in Table 2) are shown in Supplementary Figure S1.

**Table 2:** miRNAs studied and their base sequence mismatches between human and mice origin *, and references for their prognostic, predictive, or functional role in HER2^+^ BC/TNBC. Light green: no mismatch, green: 1 mismatch, dark green: 2 mismatches.

	No Mismatch—Conserved (Identical Nucleotide Sequence in Mice and Humans)	1 Mismatch	2 Mismatches	Prognostic, Predictive, Functional Role in HER2^+^/TNBC
**miR-21-5p**				[41,61,62]
**miR-26a-5p**				[41]
**miR-29a-3p**				[41,43,44]
**miR-30a-3p**				[41]
**miR-30b-5p**				[41]
**miR-34a-5p**				[41,63]
**miR-125b-5p**				[36,41]
**miR-145-5p**				[41,44,63]
**miR-148a-3p**				[41,64]
**miR-200a-3p**				[41,44]
**miR-200b-3p**				[41,44]
**miR-205-5p**				[41,44,62,65]
**miR-221-3p**				[5,41,44]
**miR-7-5p**				[66,67]
**miR-31-5p**				[68]
**miR-92a-3p**				[5,44,69]
**miR-424-5p**				[70,71]
**miR-503-5p**				[5,72]
**miR-23b-3p**				[41]
**miR-378g**				[73]
**miR-155-5p** **				[41,74]

* Base sequences of human and mice origin are given in Supplementary Material Table S2. Mismatches have been detected by sequence analysis using miRbase (http:/mirbase.org). ** “human specific” according to Guglielmi et al., 2020 [74].

Figure 1 shows conserved miRNAs, which could be detected as circulating miRNAs in sera of humanized mice transplanted with TNBC cell line MDA-MB-231, and with HER2^+^ cell line JIMT-1, and in a non-humanized mouse control (NSG). Expression levels for the three different miRNAs shown (miR-21-5p, -200a-3p, and -205-5p) appear in the same order of magnitude. miRNA expression levels changed after X-irradiation (MDA-MB231 or JIMT-1) and combined treatment (X-irradiation + PD-L1). However, at this stage of our proof-of-principle investigation, the calculation of statistical significance is not warranted, and a detailed discussion of the response and changes of miRNA expression—concerning their possible function and target(s) after treatment of TNBC (MDA-MB231)- or HER2^+^ (JIMT-1)-HTMs—would be speculative. Nevertheless, it is interesting that two miRNAs (miR-21-5p and miR-205-5p) exhibit expression levels in HTM, which are higher than those in untreated non-humanized (NSG) mice. This finding led us to speculate that these two miRNAs may be of human origin, because only transplantation with human MDA-MB-231 and JIMT-1 cell lines, along with human HSCs, distinguishes the mice from NSG controls. However, at this stage of investigation, it remains unclear whether the circulating miRNAs originate from the human tumor cells (MDA-MB-231 or JIMT-1) or from human HSCs.

Figure 2 shows detectable levels of circulating miR-503-5p (exactly one single mismatch between the mouse and human nucleotide sequence) in the serum of HTM for treated and untreated mice. Although the expression level is lower compared to miRNAs shown in Figure 1, treatment(s) seem to change the levels of miR-503-5p in both HTMs (with no proof of statistical significance and possible interpretation of function of miR-503-5p). Expression levels are higher after X-irradiation alone than those in the control HTMs. Furthermore, for all treatment conditions, miR-503-5p expression levels are remarkably higher than those in untreated non-humanized NSG mice. This is indicative for a human origin of miR-503-5p in the HTMs tested in our investigation. This interpretation is supported by an 80% probability of our detection assay (FirePlex^®^) to discriminate between human and mouse miRNA sequences in case of one nucleotide mismatch.

Figure 3 shows results for miR-23b-3p, an example of a microRNA with two mismatches between the human and mice nucleotide sequence, with approximately 95% specificity that the human miRNA can be detected by the FirePlex^®^ Assay in a mixture of mice and human circulating miRNAs in sera of HTM. For this miRNA, JIMT-1 HTMs seem to exhibit less of a response to any type of treatment compared to MDA-MB-231 HTMs (no proof of significance). Furthermore, the expression level is elevated in HTMs compared to untreated, non-humanized mice (NSG), indicating once more the human origin of miR-23b-3p (see above).

According to a well-conducted analysis by Guglielmi et al. [74], miR-155-5p cannot be detected in mice, but only in human melanoma xenografts, and was therefore classified as “human-specific”. As can be seen in Figure 3, the expression level of miR-155-5p is, overall, very low in sera (compared to all other miRNA examples, see Figure 1, Figure 2 and Figure 3). However, the expression is hardly detectable in NSG mice (dotted red line), and distinctly changed in HTMs under different treatment regiments. This underlines the ability of our assay to detect miRNAs of human origin in sera of HTM.

For all miRNAs investigated in HTMs transplanted with MDA-MB-231 or JIMT-1, it is noteworthy that approximately 59% exhibited a higher expression of miRNAs compared to untreated, non-humanized NSG mice, thus indicating that these miRNAs are possibly also of human origin (see Supplementary Figure S1). However, due to the single-mouse/-treatment character in our experimental setup, we are not able to give a detailed discussion and/or analysis of detected miRNA changes after mice treatments in this phase of our investigation.

**Figure 5:**
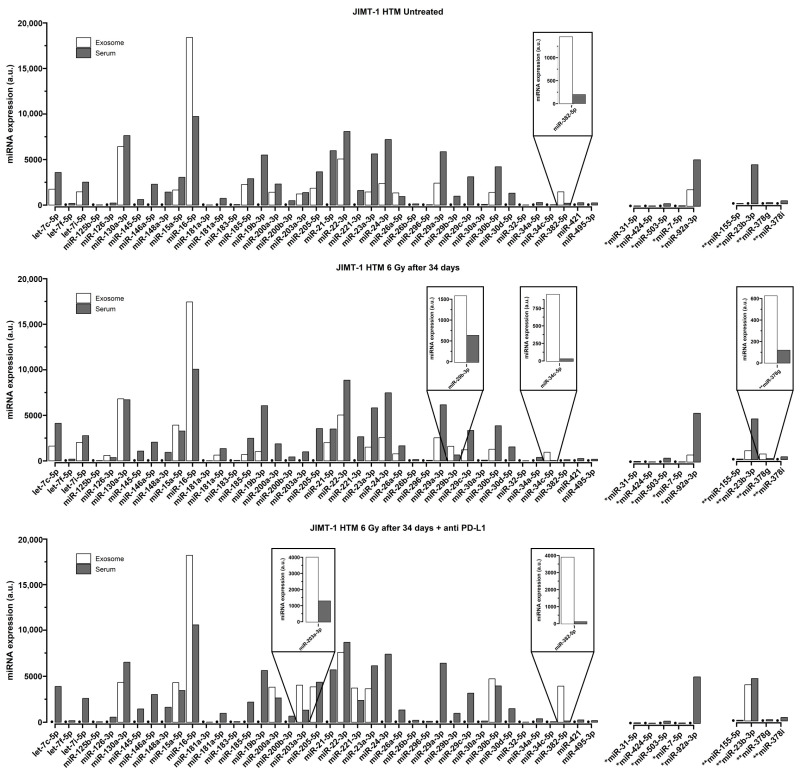
Comparison of miR expression, **serum vs. exosome**, in mice transplanted with HER2^+^ cell line (JIMT-1) after different treatments (irradiation, 6 Gy, and 6 Gy + anti-PD-L1 antibody) are shown. Higher expressed miRs in exosomes (≥2-fold, compared to serum) are highlighted by the box inserts. miR expression in exosome or serum is shown as white or black bars. miRNAs not detected in serum samples are not included. miRs not detected in exosome are indicated as follows: ●. miRNAs with mismatches are indicated with “*” for one or “**” for two mismatches. Normalizers: miR-16-5p, miR-22-3p, and miR-130a-3p.

To understand whether levels of circulating serum miRNAs might be different from the miRNA cargo of exosomes in the serum of humanized mice, we conducted an exemplary comparison (only in JIMT-1 HTM) between circulating serum miRNAs (whole panel of investigated miRNAs, see Supplementary Table S1) and the corresponding exosomal fraction (in untreated as well-treated mice). The results are shown in Figure 5.

In untreated JIMT-1 HTM controls, approximately 24% (12/49) of miRNAs were detected in the exosomal fraction, albeit at lower levels compared to free-circulating miRNAs in the serum. Other miRNAs (indicated by a dot (●) in Figure 4) seem to not be encapsulated (about 76%) in exosomes (not detectable). After 6 Gy X-irradiation in JIMT-1 HTM, about 45% (22/49) of miRNA can be found in the exosomal fraction and 55% (27/49) of miRNA seem to not be encapsulated in exosome. About 35% (17/49) of miRNAs can be found in the exosomal fraction and 65% (32/49) of miRNA seem to not be encapsulated in exosome after the 6 Gy + PD-L1 antibody treatment of JIMT-1 HTM. Interestingly, for certain miRNAs (*miR-29b-3p, miR-34c-5p, miR-203a-3p, miR-378g, and miR-382-5p* (highlighted boxes in Figure 5)), we found higher levels (≥2-fold compared to sera) of miRNA expression in exosomes of untreated mice or after different treatments of mice.

## 3. Discussion

There remains an urgent need to identify and establish additional biomarkers that enable monitoring the tumor progression and therapy response of BC. The value of eligible biomarkers can be assessed particularly in a preclinical human-like in vivo model.

In this context, circulating miRNAs in liquid biopsies have emerged as promising diagnostic, prognostic, and potentially predictive biomarker candidates [32,33,34,35]. These miRNAs can track tumor development and several treatment options, amongst them being mono- or combi-immunotherapies, as well as combinations of radio- and immunotherapies [36,37,38]. The stability of miRNAs, particularly when encapsulated in small extracellular vesicles (sEVs or exosomes), and their functional relevance, make them excellent biomarker candidates for preclinical in vitro (cell culture) and human-like in vivo (animal) studies, ultimately extending to BC patients [53,54,55,56,57].

This study presents a proof-of-principle approach using Humanized Tumor Mice (HTM) to detect human-derived miRNAs in the blood sera of mice transplanted with human BC cell lines (JIMT-1 and MDA-MB-231) as models for HER2^+^ BC and TNBC, respectively. Utilizing this model to study different treatment regimens, including immunotherapy (ICI), under preclinical conditions, required the use of mice initially transplanted with human hematopoietic stem cells to incorporate components of the human immune system.

miRNA expression was measured in liquid biopsies (sera) of HTM, which have been exposed to ionizing radiation alone (MDA-MB-231) or in a combination of irradiation and ICI using the anti-PD-L1 antibody, atezolizumab (JIMT-1). Quantitative data were compared to those derived from untreated HTM or NSG mice. Based on a FirePlex^®^ assay (Abcam), we identified and quantified 22 (most prominently detected) miRNAs in HTM sera and differentiated tumor-specific and treatment-dependent (X-ray alone or X-ray plus atezolizumab) miRNA profiles in HTM.

Our findings provide initial evidence that the level of selected circulating miRNAs in sera of HTM changes as a function of well-defined treatments. Notably, miRNAs 23b-5p and 155-5p were elevated upon X-irradiation with and without ICI treatment (anti-PD-L1) compared to untreated HTM or non-HTM. In our miRNA detection assay, human miR-23b-5p and miR-155-5p, which carry two mismatches in nucleotide sequence between human and mouse origin, are detected with 95% probability as being human-specific (see Section 4.2.2). Furthermore, miR-155-5p was classified as “human specific” in a human melanoma xenograft mouse model [74]. Both miRNAs play crucial roles in BC, which are as follows: dysregulation of miR-23b-5p is associated with trastuzumab resistance in HER2^+^ BC cells [75], while miR-155-5p has been implicated, e.g., as a predictive and prognostic biomarker for early BC recurrence and therapy resistance [76,77]. Therefore, these miRNAs exemplify that our *proof-of-principle-*approach can uncover treatment-induced changes in important, BC-associated, human miRNAs in HTMs in preclinical investigations. Our preliminary findings are promising; however, they need further validation by extended studies, especially to unequivocally show that detected (and analyzed) miRNAs are of human (tumor-) origin in HTMs.

A more definitive differentiation between human- and mouse-specific miRNAs in HTMs could be achieved by analyzing the miRNA cargo of human-specific extracellular vesicles (exosomes). We therefore isolated exosomes from sera of HTM after different treatments and enriched the exosome-specific miRNA cargo for further investigation with our flow cytometric miRNA expression assay. To this end, in a proof-of-principle investigation, we isolated extracellular vesicles from the sera of JIMT-1-transplanted HTM (HER2^+^ BC). According to their size distribution (as determined by transmission electron microscopy, TEM), vesicles had a mean diameter of about 80 nm, and their concentration ranged from 1.3–8.6 × 10^10^ particles/mL, characterizing them as exosomes, according to [78]. However, due to the low frequency of human-specific exosomes in HTM sera, we were unable to definitively confirm their human origin. The use of human-specific exosome surface markers (e.g., CD9, CD63, CD81, and HER2 for JIMT-1 HTMs) remained below the detection limit in immune fluorescence image flow cytometry . Nevertheless, it was possible to enrich miRNAs from the exosomal fraction, which enables us to compare the miRNA cargo of exosomes with free-circulating miRNAs in sera of JIMT-1 HTM.

Several miRNAs were selectively enriched in exosomes (*miR-29b-3p, miR-34c-5p, miR-203a-3p, miR-378g, and miR-382-5p*) of untreated and treated JIMT-1 HTM (Figure 5). These miRNAs show a comparably low expression in sera of HTM; however, they could be detected in exosomal fractions. They have been linked to BC treatment, BC progression, and discussed as exosomal miRNA progression-markers in liquid biopsies of BC patients [79,80,81,82].

In our investigation, at least miR-378g, selectively encapsulated in exosomes of JIMT-1 HTM sera after irradiation (6 Gy X-rays), is likely of human origin (two mismatches in sequence between mice and human), and it could be a relevant biomarker candidate for the translation of HTM results to a human BC situation. More specifically, miR-378g has a prognostic value [83] and has been shown to enhance the radiosensitivity of cancer cells [84].

At the current stage of our investigation, and depending on the available methods, it was not possible to calculate a potential statistical significance for miRNAs level changes in HTM as a function of treatment and/or as a breast cancer entity. Furthermore, due to the large sequence overlap between miRNAs of mice and human origin (no mismatches, conserved miRNA, see Table 2), the species-specific assignment remains challenging. However, we observed that ~59% of investigated miRNAs exhibited a higher expression in HTMs than in control NSG mice (Figure 2, Figure 3 and Figure 4 and Supplementary Figure S1). Furthermore, among the 13 conserved miRNAs in our study, several showed increased levels after certain days of (human) tumor treatment (X-ray and/or ICI, see Supplementary Figure S1) compared to the HTM control (without treatment). This finding indicates that even a detection of changes in levels of conserved miRNAs might deliver useful information in mouse–human models. This is in line with the findings of Guglielmi et al. [74], of a human melanoma xenograft in a mouse brain. The authors reported an increased level of conserved plasma miRNAs in mice that developed a brain tumor compared to those without a tumor. This leads us to speculate that even expression of conserved miRNAs and their treatment-induced changes observed in HTM mirror, at least partly, human tumor-specific alterations after a careful follow-up of tumor growth in HTMs in future experiments.

We are aware that our study has preliminary character and limitations. One limitation is the small number of animals, which limits the statistical significance of the data. Another limitation is the high degree of miRNA conservation in mice and men, which curbs the detection specificity for “pure” human miRNAs. However, the validity of miRNA increases with the number of mismatches (≥2), and even conserved miRNAs might be useful, considering, e.g., tumor growth and possibly other parameters for the indirect classification of miRNA origins. Limitations will be overcome in future studies using next-generation sequencing assays for miRNA identification, enabling the inclusion of other noncoding RNAs (lncRNA, as well as circular RNAs) in the analysis. This will also include a miRNA analysis of the BC cell lines used for transplantation of mice, to get basic results on their response after different treatments.

Furthermore, more sophisticated purification methods of human tumor-derived extracellular vesicles (e.g., by using microfluidic systems) will be applied. This will include a closer look (using proteomic approaches) into the human-specific protein expression on the extracellular vesicles (EV) surface to allow for more selective enrichment of human tumor-derived EVs, and for the analysis of their miRNA cargo. It will also exclude the detection of miRNAs originating from human hematopoietic stem cells, which have been used to generate HTMs. EVs can then be used to follow tumor progression, responding to different (new) therapy regimens and to the development of resistance in HTM, and to increase the reliability of the clinical translatability of certain miRNA/EV-based biomarker candidates. It will thus be the goal to compare liquid biopsies in HTM and human patient cohorts, to validate the findings and to develop monitoring assays in a clinical setting. Using liquid biopsy- (blood-, plasma-) based biomarkers, like (exosomal) miRNAS, will allow for the longitudinal tracking of tumor development (including early markers of metastasis) in patients during therapy, leading to an improvement of early therapy resistance detection and/or better timing of necessary switches in therapy regimens.

## 4. Materials and Methods

### 4.1. Humanized Mouse Model

The humanized mouse model, used in this investigation, has been extensively described elsewhere and was used to study human BC development and treatment in mice [85,86] before.

Briefly, CD34^+^ hematopoietic stem cells (HSC) were isolated from the umbilical cord blood using immunomagnetic beads (Miltenyi Biotech, Bergisch Gladbach, Germany) upon Pancoll (PAN Biotech GmbH, Aidenbach, Germany) density centrifugation. Approximately 0.1 × 10^6^ human CD34+ cells were transplanted intrahepatically in two-day old female three hours beforehand 1 Gy irradiated NOD.Cg-*Prkdc^scid^ Il2rg^tm^*^1*Wjl*^/SzJ (NSG) mice; the mice were bred and housed in a specialized pathogen-free facility (maintained at 20–24 °C, with a 12-h light/dark cycle, and 40–60% relative humidity with no restrictions to food and water) at the University of Regensburg. The generation of a human immune system was tested approximately eight weeks post-transplant in the peripheral blood. Subsequently, JIMT-1 and MDA-MB-231 tumor cells were transplanted into the inguinal mammary fat pad and tumor growth monitored twice a week. Mice were housed in small groups, nesting materials were provided, and soaked food pellets were provided if suitable. The 3Rs concept of reduce, refine, and replace was applied.

#### 4.1.1. Cell Lines Used to Induce BC in the Humanized Mouse Model

JIMT-1 (DSMZ ACC-589) and MDA-MB-231 (ATCC no. HTB-26™) cells (*developed from HER2^+^ BC and TNBC tissue*) were cultured under standard culture conditions (37 °C, 5% CO_2_) in DMEM (MDA-MB-231) or RPMI (JIMT-1) medium supplemented with 5% FCS (Thermo Fisher Scientific Inc., Waltham, MA, USA).

#### 4.1.2. Irradiation and PD-L1 Treatment of Humanized Mouse

**Figure 6:**
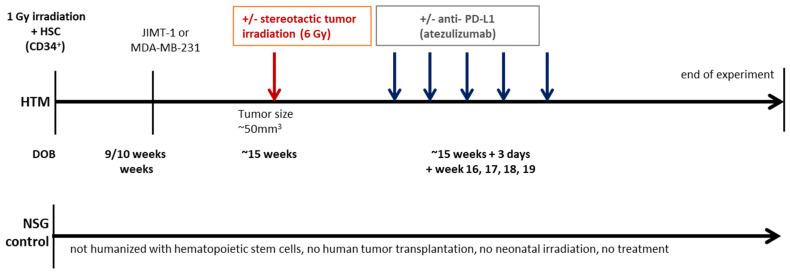
Treatment regimen of analyzed Humanized Tumor Mice (HTM). Newborn NSG pups were irradiated with 1 Gy and subsequently transplanted with 1 × 10^5^ CD34^+^cells intrahepatically. After nine to ten weeks, blood was collected, and human reconstitution was analyzed. One week later, the mice were orthotopically transplanted with JIMT-1 and MDA-MB-231 breast cancer cells. Therapy was started when tumors were palpable (~50 mm^3^). In case of irradiation, only the tumor areal was irradiated with 6 Gy. Anti-PD-L1 antibody (5 mg/kg body weight) was administered i.p. weekly, for five weeks. In the 6 Gy + anti-PD-L1 combined treatment group, anti-PD-L1 administration is started three days after irradiation. DOB = day of birth.

HTM from the same cord blood donor were divided equally in the different control and treatment groups, and treatments started when tumors reached the volume of 50 mm^3^. Local low-dose X-ray irradiation (6 Gy) was performed in the Department of Radiation Oncology (University Hospital Regensburg) using an electron linear accelerator. The anti-PD-L1 antibody atezolizumab was administered intraperitoneal (5 mg/kg) once a week for five weeks. Table 1 summarizes the treatment conditions for different HTMs. A time schedule of irradiation and anti-PD-L1 treatment is shown in Figure 6.

#### 4.1.3. Blood Harvest, Serum (Plasma) Preparation of Treated (Section 4.1.2) Mice

At the end of the experiments, mice were anaesthetized (midazolam 5 mg/kg, fentanyl 0.05 mg/kg, and medetomidine 0.5 mg/kg i.p.) and blood was collected by retroorbital puncture. Upon centrifugation (10 min, 4 °C, 10,000 rpm), serum was collected and frozen at −80 °C.

### 4.2. miRNA Detection in the Humanized Mouse Model

#### 4.2.1. miRNA Quantification in Serum of Humanized Mice

We used the flow cytometric FirePlex^®^Assay (Abcam), which enabled us to detect the amount (also mentioned as “expression level” in the following text) of up to 60 miRNAs, simultaneously, in liquid biopsies (e.g., blood serum, plasma). The assay has been successfully used in our laboratory before [87].

##### The FirePlex^®^ Assay for miRNA Profiling in Serum and Exosome

The transcription of miRNAs was measured via flow cytometric quantification of a barcode-labelled fluorescent miRNA-hydrogel-microparticle (“FirePlex Particle Technology”; FirePlex^®^ miRNA Assay V3, ab218365, with a customized miRNA focus panel, Abcam, Cambridge, MA, USA), according to the manufacturer’s protocol as described in our previous publication [87]. Briefly, a sample volume of 25 µL (protease-digested serum or exosome) was directly added to customized firefly particles (~35 µL) and incubated under shaking (e.g., 1125 rpm, see manufacturer’s protocol) at 37 °C for 60 min. After binding miRNAs to the particles, which contain complementary sequences, the miRNA-particle complexes were rinsed with rinse buffer A twice, which was followed by a labelling reaction (RT, 60 min, 1125 rpm). During the labelling reaction, each miRNA is ligated to two linkers. After washing the miR-linker-bound particles with rinse buffer B and buffer, A the miR-linkers were eluted (dissociated and released) from the particles with H2O at 55 °C. The miR-linkers were amplified by PCR and the PCR products were transferred back again to the initial particles to bind them to the complementary sequences on the particles (shaking at 1125 rpm, 37 °C, 60 min). A fluorescent reporter that binds to the miR-linker-complex was added to the particles (RT, 15 min, 1125 rpm). Fluorescence on the particles (corresponding to expression of individual miRNAs) was then measured by flow cytometry (with e.g., Guava easycyte 8HT, Millipore, Burlington, MA, USA). The raw data obtained from flow cytometry were then processed with the “FirePlex Analysis Workbench software v2.0.274” (Abcam). For the normalization, the geometric mean of the most stably expressed miRs (miR-16-5p, miR-22-3p, miR-130a-3p) was accessed based on the geNorm algorithm, with FirePlex Workbench software. The miRs were analyzed and the normalizers are given in the corresponding data tables and figures in the text. A miR expression level has been regarded as not detectable when the expression level did not reach the level of the “Limit of Detection”, which has been defined as the detection level of three off-species control miRNAs tested in the FirePlex^®^ assay (manufacturer protocol).

All of the miRNA analyses in sera and in exosomal fractions of sera in different HTMs and after different treatments were based on “single-mouse serum investigations”.

##### miRNA Selection

In this proof-of-principle investigation, we used a custom-built panel of about 54 miRNAs to analyze their expression in humanized mice blood sera. The selection of these miRNAs was hypothesis-driven, based on non-systematic literature research, identifying miRNAs involved in TNBC, HER2+ BC (cancer progression, metastasis, and inflammation, etc.). A list of miRNAs in this custom-built kit is given in Supplementary Table S1. A fraction of these miRNAs was used to investigate HTM samples (see Table 2).

#### 4.2.2. Discrimination of Human and Mice miRNAs in a Humanized Mouse Model

One of the challenges in the investigation of circulating human miRNAs in xenograft mouse models, or in a humanized mouse model (as investigated in this study), is the high degree of conserved miRNA sequences between mice and humans (see, e.g., miRbase, http://mirbase.org). This makes it difficult to discriminate between human and mouse circulating miRNAs in sera or in exosomes of humanized mice bearing human tumors. Furthermore, it is challenging to decide whether miRNA expression changes are due to developments in the human tumor/immune system, or have their origin on the mouse genomic level. Therefore, we analyzed miRNA expression changes in the sera of treated humanized mice, focusing on miRNAs that are fully **conserved** between humans and mice (no nucleotide mismatches), as well as those with **one or two mismatches** in their sequences. Additionally, we examined a miRNA (miR-155-5p), which was classified to be “**human specific**” (see Table 2). Nevertheless, according to manufacturer information (FirePlex^®,^ Abcam, Cambridge, MA, USA), the miRNA detection assay used in this investigation discriminates humans from mice miRNAs with 80% and 95% specificity in the case of 1 and 2 mismatches in the miRNA sequence. Out of the total number of microRNAs which could be tested in this investigation (see Supplementary Table S1), only those were selected (Table 2) which have already been reported to possess prognostic, predictive, or functional roles in HER2^+^ BC/TNBC.

### 4.3. sEV (Exosomes) and Their miRNA Cargo

To obtain more detailed information, especially about human-specific miRNAs in a BC-based humanized mouse model, it would be most convincing to study the miRNA cargo in human tumor-specific extracellular vesicles (EVs) or small extracellular vesicles, sEVs (exosomes) which are released into mouse blood (serum). This would require the isolation of exosomes from mouse serum based on exosome-specific lipid membrane protein markers (e.g., CD9, CD63, CD81), in combination with human tumor-specific markers which are presented on the surface of exosomes.

In our first attempt to show the possibility of detection of circulating miRNA in sera and the exosomal fraction of sera from humanized mice, we isolated exosomes (regardless of their surface markers) from mice sera by size exclusion chromatography and determined their size and number (see Section 4.3.1). Afterwards, we analyzed the miRNA cargo of the exosomal fraction using the FirePlex^®^ assay (see Section “The FirePlex^®^ Assay for miRNA Profiling in Serum and Exosome”) and compared it to the pattern of free-circulating miRNAs in mice sera.

#### 4.3.1. Isolation of Exosomes from Serum of Humanized Mice

Exosomes were isolated from the serum of humanized mice and characterized by size and concentration using Size Exclusion Chromatography (SEC), Tunable Resistive Pulse Sensing (TRPS), and Transmission Electron-Microscopy (TEM).

##### Size Exclusion Chromatography (SEC)

A total of 250 µL of serum was centrifuged at 4 °C and 15,000× *g* for 30 min to remove debris and microvesicles. The supernatant was gently removed to a new tube and filled up to 500 µL with PBS for SEC. SEC was performed using an Automatic Fraction Collector and qEV Original 35 mm SEC columns (Izon Science Ltd., Lyon, France) according to the manufacturer’s protocol. Furthermore, 500 µL fractions were collected. For determination of the size and concentration of vesicles using TRPS and the exosomal miRNA status using the Fireplex Assay, the first three fractions after void were combined.

##### Tunable Resistive Pulse Sensing (TRPS) and Electron Microscopy

The size distribution and concentration of particles in isolated exosome SEC fractions were measured using a qNano Gold TRPS system, an NP100 Nanopore, an Izon reagent kit, and CPC100 calibration beads (all Izon Science Ltd., Lyon, France), according to the manufacturer’s protocol. The diluted SEC samples (1:20) were measured at 46.5 mm stretch with a voltage of 1.08 V at two pressure levels of 6 and 9 mbar. The data were analyzed using Izon Control Suite software V3.3.2.2001.

Samples have been prepared for transmission electron microscopy (TEM) imaging using negative staining. Samples were fixated 1:4 with 4% paraformaldehyde (PFA) and 2.5% glutaraldehyde (GA) in 0.1 M cacodylate buffer (pH = 7.4). After fixation for 10 min at room temperature, 5 µL of each sample were applied with 2 min incubation to a copper grid coated with formvar and carbon (SP162, Plano, Wetzlar, Germany). All liquid was removed carefully using absorbent paper. The grid was stained three times using a droplet of 1.5% uranylacetate (UA) in aqua bidest; each was removed after 10 sec incubation. Each sample was dried at room temperature for at least 30 min after carefully removing all liquid. TEM images were taken using a JEOL JEM-2100plus (JEOL, Tokyo, Japan), at 200 kV, equipped with a Matataki Flash camera (JEOL, Tokyo, Japan).

**Figure 7:**
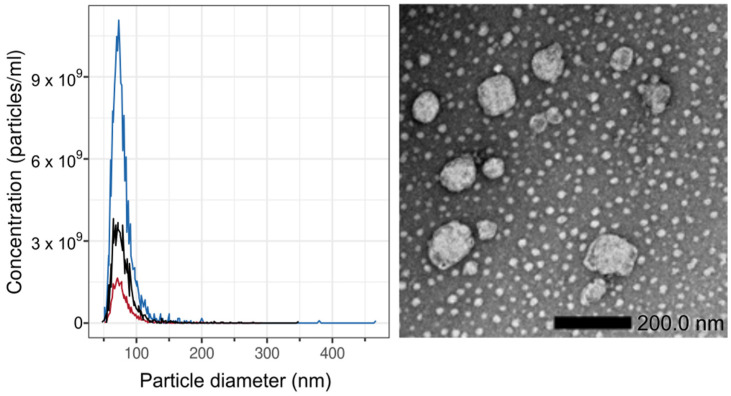
Particle (exosome) diameter (nm) distribution (left) and TEM (right) of exosome fraction after size exclusion chromatography (SEC). As can be seen, exosome diameters are in the range expected for a small extracellular vesicle (exosomes): 80 nm, regardless of treatment conditions of mice (see Table 3).

Figure 7 shows the size distribution of the exosome preparation determined by TRPS, as well as a TEM image illustrating the vesicle morphology (see above).

Further attempts to isolate human-specific exosomes from sera of HTM, by using antibodies to human-specific surface markers (e.g., human CD9, CD63, CD81, or HER2) on the exosomal lipid bilayer and immune fluorescence, as well as image flow cytometry, failed . This is probably due to the small available volumes of mice sera, the low concentration of exosomes in sera, and low fluorescence intensities after immune staining.

## 5. Conclusions

This proof-of-principle investigation demonstrates for the first time that circulating miRNAs can be detected in sera of mice in an HTM model, which uses transplantation of human BC cell lines. The animal models allow us to evaluate the regulation of miRNAs, as well as exosomes and their cargos, as a function of treatments, e.g., irradiation, immune checkpoint inhibition, and a combination of both. Importantly, it was also shown that selected miRNAs are enriched in exosomes purified from HTM sera.

Extended studies are required to further validate the HTM model for the qualitative and quantitative analysis of free-circulating miRNAs and/or extracellular nucleic acids that have the capacity to control human tumor growth and progression, as well as an immunological tumor defense. Ultimately, this model could serve as a platform to discover and characterize miRNA-based diagnostic, prognostic, and predictive biomarkers for monitoring (neoadjuvant) treatment options in clinical settings.

## Figures and Tables

**Table 1:** Treatment of humanized mice (JIMT-1, MDA-MB-231-transplanted).

Mice	Cord Blood Donor	Transplanted Cells	Tumor Volume ~ 50 mm^3^ (Days Post Tumor Transplant)	Treatment of Mice (Days Post Tumor Transplant)	Isolation of
Her2^+^ HTM	CB donor 7	JIMT-1	30	6 Gy day 34	Serum miRNA, Exosomal miRNA
Her2^+^ HTM	CB donor 7	JIMT-1	34	6 Gy day 34 + anti-PD-L1	Serum miRNA, Exosomal miRNA
Her2^+^ HTM	CB donor 7	JIMT-1	37	No treatment	Serum miRNA, Exosomal miRNA
TNBC HTM	CB donor 10	MDA-MB-231	50	6 Gy day 55	Serum miRNA
TNBC HTM	CB donor 10	MDA-MB-231	71	6 Gy day 55	Serum miRNA
TNBC HTM	CB donor 10	MDA-MB-231	66	No treatment	Serum miRNA
NSG (control)		No		No treatment	Serum miRNA

All HTM transplanted with JIMT-1 were derived from cord blood donor 7; those transplanted with MDA-MB-231 were derived from donor 10 (all mice in each group received the same cord blood donor).

**Table 3:** Characteristics of exosomes isolated from different sera of humanized mice after or without irradiation (+/−), and with and without immune checkpoint therapy (ICI +/−).

SERUM	IRRADIATED *	ICI *	PARTICLES/ML	MEAN DIAMETER [NM]
HER2+ HTM, JIMT-1	+	−	1.3 × 10^10^	80
HER2+ HTM, JIMT-1	+	+	8.6 × 10^10^	78
HER2+ HTM, JIMT-1	−	−	3 × 10^10^	80

* s. is also shown in Table 1.

## Data Availability

The data that support the findings of this study, as well as the R code used for the analysis, are publicly available at https://github.com/MBender1992/BREAST_REG.

## Supplementary Materials

The supporting information can be downloaded at: https://www.mdpi.com/article/10.3390/ijms26083629/s1.
