# Supplementary material for: Detection of Human Circulating and Extracellular Vesicle-Derived miRNAs in Serum of Humanized Mice Transplanted with Human Breast Cancer (HER2+ and TNBC) Cells—A Proof of Principle Investigation
Source: Int J Mol Sci. 2025 Apr 11;26(8):3629. doi: 10.3390/ijms26083629 (PMC12026515; doi:10.3390/ijms26083629)
Supplement: Supplementary file 1 [file ijms-26-03629-s001.zip › ijms-3496078-supplementary.pdf]

Supplementary material

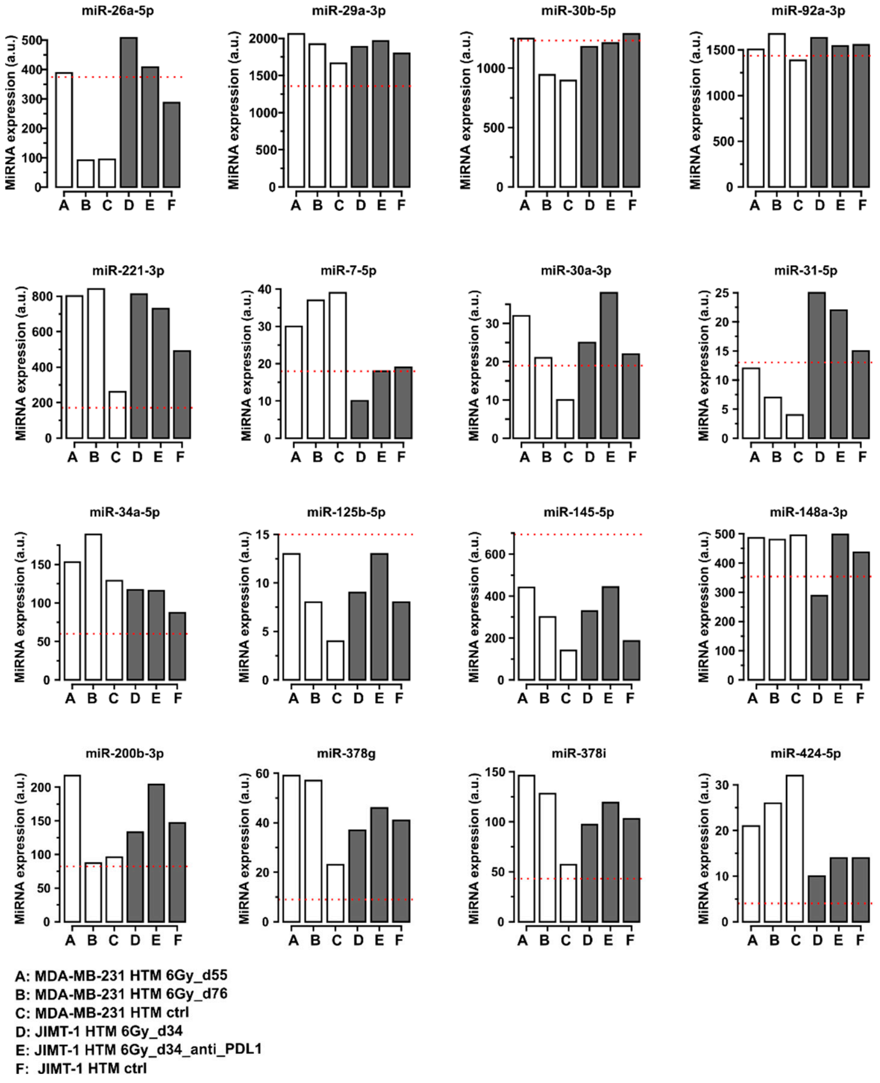

Supplementary Figure S1 *Circulating miRs in serum of humanized mice (HTM). Results from mice transplanted with TNBC cell line MDA-MB-231 and those transplanted HER2+ cell line (JIMT-1) are shown after different treatments (irradiation, 6Gy, and 6Gy + PD-L1-antibody, azetolimab, (JIMT-1)). miRNA expression levels for the miRNAs is furthermore compared with those levels in serum of un-treated HTM controls and un-treated NSG-mice, dotted red line.* Time points after cell line transplantation are given in dxy. *miRNA levels have been normalized to geometrical mean of expression of most stable miRNAs 16-5p, - 22-3p, and -130a-3p in FirePlex Assay (s. 2.2.1.1). Expression values of miRNA. are given in arbitrary units (AU) of fluorescence intensity values in the flow cytometric FirePlex Assay*.

Supplement Tables

Supplementary Table S1

List of miRNAs studied				
hsa-let-7c-5p	** hsa-mir-23b-3p	hsa-mir-32-5p	hsa-mir-148a-3p	hsa-mir-296-5p
hsa-let-7f-5p	hsa-mir-24-3p	hsa-mir-34a-5p	** hsa-mir-155-5p	** hsa-mir-378g
hsa-let-7i-5p	hsa-mir-26a-5p	hsa-mir-34c-5p	hsa-mir-181a-3p	** hsa-mir-378i
* hsa-mir-7-5p	hsa-mir-26b-5p	* hsa-mir-92a-3p	hsa-mir-181a-5p	hsa-mir-382-5p
hsa-mir-9-5p	hsa-mir-29a-3p	hsa-mir-101-3p	hsa-mir-183-5p	hsa-mir-383-5p
hsa-mir-15a-5p	hsa-mir-29b-3p	hsa-mir-125b-5p	hsa-mir-185-5p	hsa-mir-421
hsa-mir-16-5p	hsa-mir-29c-3p	hsa-mir-126-3p	hsa-mir-200a-3p	* hsa-mir-424-5p
hsa-mir-19b-3p	hsa-mir-30a-3p	hsa-mir-130a-3p	hsa-mir-200b-3p	hsa-mir-495-3p
hsa-mir-21-5p	hsa-mir-30b-5p	hsa-mir-135b-5p	hsa-mir-203a-3p	* hsa-mir-503-5p
hsa-mir-22-3p	hsa-mir-30d-5p	hsa-mir-145-5p	hsa-mir-205-5p	
hsa-mir-23a-3p	* hsa-mir-31-5p	hsa-mir-146a-5p	hsa-mir-221-3p	

*: One mismatch; **: Two mismatches (between human and mice sequence).

Supplementary Table S2

miR sequence comparison Human (hsa) vs mouse (mmu)

**one mis-match**	
hsa-miR-7-5p	UGGAAGACUAGUGAUUUUGUUGUU
mmu-miR-7a-5p	UGGAAGACUAGUGAUUUUGUUGU
hsa-miR-31-5p	AGGCAAGAUGCUGGCAUAGCU
mmu-miR-31-5p	AGGCAAGAUGCUGGCAUAGCUG
hsa-miR-92-3p	UAUUGCACUUGUCCCGGCCUGU
mmu-miR-92a-3p	UAUUGCACUUGUCCCGGCCUG
hsa-miR-424-5p	CAGCAGCAAUUCAUGUUUUGAA
mmu-miR-322-5p	CAGCAGCAAUUCAUGUUUUGGA
hsa-miR-503-5p	UAGCAGCGGGAACAGUUCUGCAG
mmu-miR-503-5p	UAGCAGCGGGAACAGUACUGCAG

**two mis-matches**	
hsa-miR-23b-3p	AUCACAUUGCCAGGGAUUACCAC
mmu-miR-23b-3p	AUCACAUUGCCAGGGAUUACC
hsa-miR-155-5p*	UUAAUGCUAAUCGUGAUAGGGGUU
mmu-miR-155-5p	UUAAUGCUAAUUGUGAUAGGGGU
hsa-miR-378g	ACUGGGCUUGGAGUCAGAAG
mmu-miR-378c	ACUGGACUUGGAGUCAGAAGC
hsa-miR-378i	ACUGGACUAGGAGUCAGAAGG
mmu-miR-378c	ACUGGACUUGGAGUCAGAAGC

*Mismatches are indicated with underline and red; *human-specific [78]*
